# Comparison of treatment outcomes in patients with and without diabetes mellitus attending a multidisciplinary cardiovascular prevention programme (a retrospective analysis of the EUROACTION trial)

**DOI:** 10.1186/s12872-015-0006-4

**Published:** 2015-02-24

**Authors:** Sandra N Ofori, Kornelia Kotseva

**Affiliations:** Department of Internal Medicine, University of Port Harcourt Teaching Hospital, Choba, Rivers state, Nigeria; Cardiovascular Medicine, International Centre for Circulatory Health, National Heart and Lung Institute, Imperial College London, London, United Kingdom

**Keywords:** Coronary disease, Diabetes mellitus, Risk factors

## Abstract

**Background:**

The objective was to compare the improvements in lifestyle and risk factor profiles in patients with and without diabetes mellitus (DM) in the intervention arm of EUROACTION study.

**Methods:**

This was a retrospective analysis of the intervention arm of EUROACTION trial. Primary outcome was proportions meeting the European targets for not smoking, diet, physical activity (PA), body mass index (BMI), waist circumference (WC), blood pressure (BP), total and low-density lipoprotein (LDL) cholesterol and cardio-protective drug use at one year.

**Results:**

179 and 777 coronary patients with and without DM, and 340 and 917 high-risk individuals (HRI) with and without DM, respectively were identified. The proportions of coronary patients achieving the lifestyle targets improved from the initial assessment (IA) except non-smoking, which reduced. At one year, significantly fewer patients with DM attained the targets for BMI (13.2% vs 31.3%, p = 0.002) and BP <140/90 mmHg (53.5% vs 74.0%, p < 0.001) compared to patients without DM despite a higher proportion of patients with DM prescribed angiotensin converting enzyme inhibitors/angiotensin receptor blockers (79.1% vs 65.3%, p = 0.021).

Among the HRIs, fewer patients with DM achieved targets for oily fish intake (9.3% vs 11.9%, p = 0.043), physical activity (65.8% vs 75.8%, p = 0.011), and BMI (9.9% vs 28.1%, p = 0.022) at one year. While more patients with DM achieved the targets for total cholesterol (48.2% vs 22.9%, p < 0.001) and LDL (57.9% vs 30.7%, p < 0.001).

**Conclusions:**

Multidisciplinary intervention had a beneficial effect on several cardiovascular risk factors in both patients with and without DM. Poorer achievement of mostly lifestyle (and BP in coronary patients) targets among those with DM emphasises the need for more intensive lifestyle modification and BP management for the prevention of cardiovascular disease.

**Electronic supplementary material:**

The online version of this article (doi:10.1186/s12872-015-0006-4) contains supplementary material, which is available to authorized users.

## Background

Diabetes mellitus (DM) is an important risk factor for cardiovascular disease (CVD). Type 2 diabetes (T2DM) accounts for about 90% of all cases [1]. Individuals with DM have a two to four-fold higher risk of coronary heart disease (CHD) and in turn, CVD accounts for more than half of the deaths in this population [2,3]. Sedentary lifestyle, obesity, cigarette smoking, hypertension and dyslipidaemia are independent CVD risk factors that are commonly associated with DM further increasing their absolute risk thus the treatment targets for patients with DM compared to those without are stricter [4,5]. Results from the EUROASPIRE surveys illustrated poor risk factor management of coronary patients in clinical practice, which was worse among patients with DM [6-9]. However the benefit of an intensive, multifactorial intervention in high-risk T2DM patients has been demonstrated where this approach reduced the risk of macrovascular and microvascular events by 50% [10].

Following the EUROASPIRE surveys, the EUROACTION, a cluster randomised controlled trial (RCT) was carried out in 24 hospitals and general practices in eight European countries. Patients with established coronary disease and individuals at high multifactorial risk for CVD received EUROACTION intervention or usual care. The intervention was a nurse-coordinated, multidisciplinary family-based 16-week cardiovascular prevention (and rehabilitation for coronary patients) programme aimed at achieving the lifestyle, risk factor and treatment goals as defined in the 1998 Joint European Societies’ guidelines (Additional file 1: Table S1) [11]. Included among this cohort were people with DM. This present study was carried out to compare patients with and without DM in terms of achieving the targets given the same level of intervention. The primary outcome was the proportion of patients in both groups achieving the lifestyle and risk factor targets for CVD prevention at one-year follow-up (Additional file 2: Table S2). Secondary outcome was the change in proportions achieving these targets, between initial assessment and one year.

### Subjects and methods

#### Study population

The study population of EUROACTION has been described in previous publications [11,12]. Briefly, twelve (six pairs) general hospitals and twelve (six pairs) general practice (GP) centres across eight European countries were cluster randomised to receive the EUROACTION intervention or usual care. Patients with established coronary disease were recruited in general hospitals and high-risk individuals without coronary disease were recruited in general practices.

## Methods

The protocols and methods used in EUROACTION have been described previously [11,12]. The eligible patients were assessed by a multidisciplinary team at baseline for lifestyle, medical risk factors and cardio-protective drug use. Smoking status was recorded as smoker or non-smoker in the month prior to event (hospital) or interview (GP). Self-reports were validated by breath carbon monoxide (<6 parts per million consistent with non-smoking) using a Smokerlyser (Bedfont micro-smokerlyser, Bedfont Scientific, Model EC 50 Micro III). Food intake was assessed via a structured interview with a food-habit questionnaire and validated against a 7-day diet diary. Data on physical activity was collected with a 7-day activity recall diary. Weight and height were measured with standardised equipment (Seca 707 digital scales with measuring stick), and body mass index (BMI) was calculated using the formula weight (kg)/height (m^2^). The normal range is 18.5–24.9 kg/m^2^. Standardised methods were used to measure the waist circumference and values less than 102 cm for men, and less than 88 cm for women were considered normal. Total cholesterol, HDL, triglycerides, glucose, and HbA1c concentrations were analysed at a central laboratory with standardised methods and equipment [11,12].

Fasting and random glucose were measured and OGTT was performed if the fasting glucose was above 6.1 mmol/l to diagnose diabetes or impaired glucose tolerance. The study participants thereafter attended at least eight sessions conducted weekly. On completion of the 16-week programme, coronary patients were re-invited for assessment and at one year, all the patients were again reassessed.

### Sample size

For the current study, all the patients (not their partners/families) in the EUROACTION intervention arm were identified. There were 1587 coronary patients, 956 of whom had baseline FPG results. They were categorized as having diabetes or not based on a history of known diabetes and/or FPG ≥ 7 mmol/l (DM 179, non-DM 777). Out of 1257 general practice patients, 340 had an existing diagnosis of DM while 917 were not diabetic.

#### Statistical analyses

The data was analysed with STATA 12 software and the results are presented using tables and figures. Continuous variables are presented as mean (standard deviation) for normally distributed data, median (interquartile) for non-parametric data, and compared with Student’s t-tests or Mann–Whitney tests as considered appropriate. Categorical data are presented as proportions (percentages) and compared with the Chi square test at baseline. Comparisons of change from the initial assessment to one-year within each group (DM/Non-DM) were made using the paired *t*-test for continuous data and McNemar’s test for paired proportions. Since the original EUROACTION trial was cluster-randomised, individual level assessments at baseline revealed significant differences between the diabetic and non-diabetic individuals. These were taken into account in the multiple logistic regression analysis that was used to determine the odds (and 95% CI) of achieving each target given the diabetes status controlling for age and the endpoint of interest at baseline as covariates. Two-sided P values <0.05 were considered statistically significant.

#### Ethical considerations

The patients already gave written informed consent in the original EUROACTION TRIAL where the local ethics committee in each centre granted ethical approval [12] therefore this current study did not require ethical approval as it involved analysis of anonymised data already collected.

## Results

Table 1 shows the age and sex distribution of the population.

**Table 1:** **Age and sex distribution of the study population**

	**Hospital n = 956**		**General practice n = 1257**	
	**DM**	**Non-DM**	**DM**	**Non-DM**
**Age group (years)**				
<55	31 (17.3)	236 (30.4)	69 (20.3)	279 (30.4)
55–64	72 (40.2)	266 (34.2)	132 (38.8)	397 (43.3)
≥65	76 (42.5)	275 (35.4)	139 (40.9)	241 (26.3)
**Sex**				
Male	119 (66.5)	567 (72.9)	164 (48.2)	471 (51.4)
Female	60 (33.5)	210 (27.1)	176 (51.8)	446 (48.6)
Total	179 (18.7)	777	340 (27.0)	917

Data presented as number (percentage); DM-diabetes mellitus.

### Characteristics of the study population at initial assessment

At baseline among the coronary patients, the diabetics were significantly older (Table 2). There was overall sub-optimal adherence to lifestyle recommendations. Fewer diabetics met the target for saturated fat compared to non-diabetics. Diabetics had significantly higher mean weight, WC and BMI and the proportions achieving the targets for WC and BMI were significantly lower when compared to non-diabetics. Mean TC and LDL were lower and the proportion at target LDL was significantly higher among diabetics. On the other hand, SBP was significantly higher and the proportion of diabetics at target BP was significantly lower compared to the non-diabetics. A high proportion of diabetics and non-diabetics used cardioprotective drugs. Only the use of ACEI/ARB was significantly higher among diabetics compared to non-diabetics.

**Table 2:** **Proportion of coronary patients and high-risk individuals meeting the targets at initial assessment**

	**Coronary patients**			**High-risk individuals**		
	**DM**	**Non-DM**	**P value**	**DM**	**Non-DM**	**P value**
**Age yrs. (mean, SD)**	62.5 (9.5)	59.8 (10.4)	0.002	62.8 (8.0)	59.9 (7.6)	<0.001
**% Female**	60/179 (33.5)	210/777 (27.0)	0.082	176/340 (51.8)	446/917 (48.6)	0.324
**% Not smoking**	169/179 (94.4)	718/777 (92.4)	0.350	277/339 (81.7)	489/772 (63.3)	<0.001
**SF target**	5/24 (20.8)	53/108 (49.1)	0.012			
**Oily fish target**	6/130 (4.6)	27/ 607 (4.5)	0.933	19/333 (5.7)	36/762 (4.7)	0.494
**Fish target**	102/179 (56.9)	416/776 (53.6)	0.414	214/333 (64.3)	457/764 (59.8)	0.165
**Fruit/veg target**	93/179 (51.9)	354/776 (45.6)	0.126	200/333 (60.1)	349/763 (45.7)	<0.001
**Physical Activity**	39/178 (21.9)	205/775 (26.5)	0.211	95/333 (28.5)	218/762 (28.6)	0.386
**Waist circum. (cm)**						
**● Mean (SD)**	101.5 (11.4)	95.9 (11.5)	<0.001	102.2 (12.7)	95.3 (12.9)	<0.001
**% At target:**						
**≤80 cm women**	5/59 (8.5)	45/209 (21.5)	0.023	8/176 (4.6)	86/446 (19.3)	<0.001
**≤94 cm men**	27/118 (22.9)	207/566 (36.6)	0.004	32/164 (19.5)	147/471 (31.2)	0.004
**BMI (kg/m** ^**2**^ **)**						
**● Mean (SD)**	29.9 (4.8)	27.8 (4.1)	<0.001	30.8 (4.8)	28.3 (4.7)	<0.001
**● % At target**	20/178 (11.2)	196/775 (25.3)	<0.001	26/340 (7.7)	183/917 (19.9)	<0.001
**TC (mmol/l)**						
**● Mean (SD)**	4.36 (1.0)	4.60 (1.1)	0.009	5.5 (0.1)	6.1 (0.03)	<0.001
**● % At target**	133/179 (74.3)	531/777 (68.3)	0.118	96/340 (28.2)	79/917 (8.6)	<0.001
**LDL (mmol/l)**						
**● Mean (SD)**	2.56 (0.9)	2.77 (0.9)	0.014	3.3 (0.1)	3.8 (0.03)	<0.001
**● % At target**	116/161 (72.1)	428/676 (63.3)	0.037	108/340 (31.8)	114/917 (12.4)	<0.001
**HDL (mmol/l) mean (SD)**	1.09 (0.3)	1.20 (0.4)	0.002	1.4 (0.4)	1.5 (0.4)	<0.001
**TG (mmol/l) mean, (SD)**	1.59 (0.8)	1.44 (0.9)	0.059	1.8 (0.1)	1.6 (0.03)	0.001
**SBP mmHg**						
**mean (SD)**	131.7 (18.6)	127.1 (18.1)	0.005	140.3 (18.5)	141.1 (18.7)	0.520
**DBP mmHg mean (SD)**	74.5 (10.8)	75.7 (10.7)	0.204	81.5 (9.5)	85.4 (10.9)	<0.001
**BP target <140/90 mmHg**	95/149 (63.8)	518/690 (75.1)	0.005	158/340 (46.5)	324/917 (35.3)	<0.001
**BP target <130/85 mmHg**	61/149 (40.9)	383/690 (55.5)	0.001	82/340 (24.1)	170/917 (18.5)	<0.001
**BB**	141/165 (85.5)	609/734 (82.9)	0.438	66/170 (38.8)	81/640 (12.7)	<0.001
**ACE/ARB**	116/166 (69.9)	407/685 (59.2)	0.013	136/224 (60.7)	56/668 (23.4)	<0.001
**Anti-platelets**	171/177 (96.6)	735/766 (95.9)	0.685	33/147 (22.5)	53/633 (8.4)	<0.001
**STATINS**	144/168 (85.7)	607/721 (84.2)	0.623	81/188 (43.0)	85/646 (13.2)	<0.001

Data are n/N (%) unless otherwise stated; ACEI/ARB-angiotensin converting enzyme inhibitor/angiotensin II receptor blocker; BB- beta-blocker; BMI-body mass index; CCB-calcium channel blocker; DBP- diastolic blood pressure; HDL-high-density lipoprotein cholesterol; LDL- low-density lipoprotein cholesterol; SBP-systolic blood pressure; SD-standard deviation; SF- saturated fat; TC- total cholesterol; TG- triglyceride.

Among the high-risk individuals, diabetics were older and significantly more were non-smokers. While only a small proportion met the target for oily fish, significantly more diabetics were at target for fruit/vegetables. Diabetics had significantly higher mean weight, WC and BMI and a lower proportion of them were at the recommended targets compared to the non-diabetics. The mean TC, LDL and HDL were significantly lower while triglycerides were higher among diabetics. Only 28.2% of diabetics achieved TC target, 31.8% LDL target and 24.1% BP target but these were significantly higher than the proportion of non-diabetics. Although less than half of the diabetics used the various classes of cardioprotective drugs (except ACEI/ARBs), the proportions were significantly higher than among the non-diabetics.

### Comparison of the proportions achieving the targets at one year and change from initial assessment

#### Coronary patients

- **Lifestyle factors:** among those who reported smoking in the month prior to their cardiac event, the odds of being a smoker at one year was 34% higher for diabetics compared to non-diabetics. In both groups (DM/Non-DM), the proportion of non-smokers significantly reduced from the IA to one-year and this difference was more among the diabetics (Table 3). With regards to the diet there was no significant difference in the change in proportions achieving these targets in both patient groups. At initial assessment, only 21.9% of diabetics and 26.5% of non-diabetics achieved PA targets but these increased significantly in both groups by one year to 48.6% and 57.5% respectively (Table 3). The proportion of diabetics achieving the BMI target was significantly lower (13.2% vs 31.3% p = 0.002).

**Table 3:** **Changes in the proportion of coronary patients achieving the targets between the initial and one year assessments**

	**DM**				**NON-DM**			
	**IA**	**1 year**	**Diff %**	**P value**	**IA**	**1 year**	**Diff %**	**P value**
**Not smoking**	138/145 (95.2)	127/145 (87.6%)	−7.6%	0.001	641/683 (93.9)	608/683 (89.0)	−4.8%	<0.001
**SF target**	5/19 (26.3)	9/19 (47.4)	21.1%	0.219	46/96 (47.9)	61/96 (63.5)	15.6%	0.011
**Oily fish target**	5/97 (5.2)	24/97 (24.7)	19.6%	0.0003	24/521 (4.6)	102/521 (19.6)	14.9%	<0.001
**Fish target**	102/179 (56.9)	121/179 (67.6)	10.6%	0.003	416/776 (53.6)	531/776 (68.4)	14.8%	<0.001
**F/V target**	78/144 (54.2)	114/144 (79.2)	25.0%	<0.001	331/683 (48.5)	501/683 (73.4)	24.9%	<0.001
**PA target**	35/144 (24.3)	70/144 (48.6)	24.3%	<0.001	192/680 (28.2)	391/680 (57.5)	29.3%	<0.001
**Ideal WC Females**	4/49 (8.2)	5/49 (10.2)	2.0%	0.317	39/184 (21.2)	48/184 (26.1)	4.9%	0.064
**Ideal WC Males**	22/94 (23.4)	30/94 (31.9)	8.5%	0.022	179/497 (36.0)	210/497 (42.3)	6.2%	0.001
**Mean weight**	81.6	82.0	0.45	0.174	79.3	78.6	−0.67	<0.001
**BMI target**	18/144 (12.5)	19/144 (13.2)	0.7%	1.000	171/677 (25.3)	212/677 (31.3)	6.1%	<0.001
**TC target**	102/137 (74.5)	101/137 (73.7)	−0.7%	1.000	464/661 (70.2)	490/661 (74.1)	3.9%	0.055
**Mean TC**	4.36 (1.0)	4.44 (1.0)	0.07	0.418	4.54 (1.1)	4.49 (1.0)	−0.05	0.213
**LDL target**	86/124 (69.4)	90/124 (72.6)	3.2%	0.627	376/585 (64.3)	416/585 (71.1)	6.8%	0.005
**Mean LDL**	2.62 (0.9)	2.64 (0.9)	0.02	0.861	2.75 (0.0)	2.61 (0.9)	−0.13	0.002
**BP target <130/85 mmHg**	58/140 (41.4)	56/140 (40.0)	−1.4%	0.880	361/645 (55.9)	332/645 (51.5)	−4.5%	0.041
**Mean SBP**	131.7 (18.8)	136.2 (21.5)	4.5	0.005	127.0 (18.0)	128.5 (17.8)	1.5	0.022
**Mean DBP**	74.2 (10.9)	75.3 (11.2)	1.1	0.207	75.7 (10.6)	76.1 (10.4)	0.4	0.237
**BB**	106/125 (84.8)	106/125 (84.8)	0%	1.000	513/615 (83.4)	510/615 (82.9)	−0.5%	0.807
**ACEI/ARB**	89/129 (68.9)	101/129 (78.3)	9.3%	0.008	346/573 (60.4)	370/573 (64.6)	4.2%	0.005
**Anti-platelet**	134/139 (96.4)	133/139 (95.7)	−0.7%	1.000	631/658 (95.9)	637/658 (96.8)	0.9%	0.308
**Statins**	113/129 (87.6)	117/129 (90.7)	3.1%	0.344	527/620 (85.0)	577/620 (93.1)	8.1%	<0.001

Data are n/N (%) proportion as a percentage (of the numbers in each group who had both IA and one year data for the variables in question) and difference between IA and one year; ACEI/ARB- angiotensin converting enzyme inhibitor/angiotensin receptor blocker; BB- beta blocker; BMI- body mass index; CCB- calcium channel blocker; DBP- diastolic blood pressure; LDL- low density lipoprotein cholesterol; SBP- systolic blood pressure; SF- saturated fat; TC- total cholesterol; TG- triglyceride.
- **Lipid and BP indices:** Although at one year similar proportions of diabetics and non-diabetics achieved the targets for TC and LDL, improvement for LDL targets from baseline occurred only among the non-diabetics (Table 3). Mean SBP increased significantly from IA to one year among diabetics and non-diabetics, however this increase was greater among the diabetics (Table 3). In addition, they were less likely to achieve the BP target <140/90 mmHg compared to the non-diabetics (OR 0.47, 95% CI 0.31-0.72; p < 0.001). With the lower BP threshold <130/85 mmHg even fewer diabetics achieved this target (36.9% vs 51.3%) but after adjusting for baseline covariates this was not significant (Table 4).

**Table 4:** **Proportions of coronary patients and high-risk individuals achieving the targets at one year according to DM status**

	**Coronary patients**				**High-risk individuals**			
	**DM**	**Non-DM**	**Adjusted OR (95% CI)** ^**a**^	**P value**	**DM**	**Non-DM**	**Adjusted OR (95% CI)** ^**a**^	**P value**
Non-Smokers*	22/38 (57.9)	111/180 (61.7)	1.34 (0.69, 2.61)	0.388	271/317 (85.5)	486/712 (68.3)	0.55 (0.29, 1.07)	0.077
**SF target**	25/51 (49.0)	153/262 (58.4)	0.50 (0.16, 1.57)	0.237				
**Oily fish target**	28/125 (22.4)	118/621 (19.0)	1.42 (0.85, 2.37)	0.185	29/313 (9.3)	84/709 (11.9)	0.58 (0.34, 0.98)	0.043
**Fish target**	121/179 (67.6)	531/776 (68.4)	0.79 (0.51, 1.22)	0.287	257/334 (76.9)	567/774 (73.3)	1.07 (0.74, 1.54)	0.723
**Fruit/Veg target**	114/144 (79.2)	501/683 (73.4)	1.34 (0.82, 2.18)	0.237	263/317 (82.9)	538/714 (75.4)	1.18 (0.80, 1.73)	0.395
**Phy. Activity target**	70/144 (48.6)	391/680 (57.5)	0.73 (0.50, 1.07)	0.103	206/313 (65.8%)	536/707 (75.8)	0.68 (0.49, 0.92)	0.011
**Ideal WC Females**	5/49 (10.2)	48/184 (26.1)	0.45 (0.12, 1.72)	0.241	13/161 (8.1)	98/344 (28.5)	0.48 (0.21, 1.07)	0.072
**Ideal WC Males**	30/95 (31.6)	210/497 (42.3)	0.83 (0.45, 1.52)	0.550	39/146 (26.7)	157/360 (43.6)	0.65 (0.36, 1.17)	0.151
**BMI target**	19/144 (13.2)	212/677 (31.3)	0.31 (0.15, 0.65)	0.002	31/313 (9.9)	198/706 (28.1)	0.49 (0.26, 0.90)	0.022
**TC <5 mmol/l**	101/137 (73.7)	490/661 (74.1)	0.79 (0.50, 1.25)	0.320	143/297 (48.2)	155/678 (22.9)	2.49 (1.82, 3.41)	<0.001
**LDL <3 mmol/l**	93/126 (73.0)	419/590 (71.0)	0.93 (0.59, 1.47)	0.770	168/290 (57.9)	205/668 (30.7)	2.43 (1.78, 3.33)	<0.001
**BP <140/90 mmHg**	77/144 (53.5)	502/678 (74.0)	0.47 (0.31, 0.72)	<0.001	210/313 (67.1)	470/707 (66.5)	0.91 (0.66, 1.	0.546
**BP <130/85 mmHg**	56/144 (38.9)	348/678 (51.3)	0.78 (0.52, 1.17)	0.234	120/313 (38.4)	242/707 (34.2)	1.16 (0.84, 1.60)	0.357
**BB**	111/130 (85.4)	525/630 (83.3)	1.10 (0.56, 2.16)	0.774	72/139 (51.8)	107/513 (20.9)	0.83 (0.34, 2.03)	0.688
**ACE/ARB**	106/134 (79.1)	384/588 (65.3)	2.11 (1.11, 3.99)	0.021	164/217 (75.6)	223/546 (40.8)	0.84 (0.43, 1.64)	0.605
**Anti-platelets**	134/140 (95.7)	644/665 (96.8)	0.65 (0.22, 1.88)	0.422	63/134 (47.0)	74/496 (14.9)	2.04 (0.90, 4.61)	0.087
**Statins**	123/135 (91.1)	603/646 (93.3)	0.55 (0.26, 1.17)	0.121	147/209 (70.3)	236/586 (40.3)	1.46 (0.94, 2.27)	0.088

**Figure 1:**
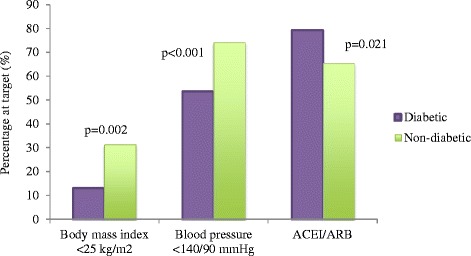
**Summary of significant differences among diabetic and non-diabetic coronary patients at one-year assessment.**

Data are n/N (%); ^a^odds ratios adjusted for age and variable at baseline (95% confidence intervals). *also adjusted for smoking in the month prior to the event; ACEI/ARB- angiotensin converting enzyme inhibitor/angiotensin receptor blocker; BB- beta blocker; BMI- body mass index; CCB- calcium channel blocker; DBP- diastolic blood pressure; LDL- low density lipoprotein cholesterol; SBP- systolic blood pressure; SF- saturated fat; TC- total cholesterol; TG- triglyceride.
- **Drug therapy:** At one year, the diabetics were significantly more likely to be on ACEI/ARB (OR 2.11, 95% CI 1.12-3.99; P = 0.021) but the proportions using other cardioprotective drugs did not differ significantly from the non-diabetics (Table 4). The use of ACEI/ARB increased significantly in both groups of patients from IA but this increase was higher among the patients with diabetes (difference 9.3% vs 4.2%). No significant changes were noted in the use of beta-blockers and anti-platelets in both groups from IA to one year. Statin use increased significantly among patients without diabetes only. This may explain the significant increase in proportions of patients without diabetes meeting the LDL targets at one year (Table 3). The significant differences among the patients with and without diabetes at one year are summarised in Figure 1.

#### High-risk individuals

- **Lifestyle factors:** in both groups, the proportion of non-smokers increased significantly from baseline (DM: difference 2.8% p = 0.035; non-DM: difference 3.4% p = 0.0003) (Table 5). Only a very small proportion of patients with diabetes achieved the target for oily fish intake and this was significantly less compared to the patients without diabetes (9.3% versus 11.9%; p = 0.043) (Table 4). In both groups, the increase in proportions achieving dietary and PA targets from baseline was significant (Table 5). However, at one year, the patients with diabetes were significantly less likely to achieve PA target compared to the patients without diabetes (Table 4). In both groups, the proportions achieving the target for WC and BMI increased slightly at one year. Only 9.9% of diabetics met the BMI target compared to 28.1% of patients without diabetes (p = 0.022).

**Table 5:** **Changes in the proportion of high-risk individuals with and without diabetes achieving the targets between the initial and one year assessments**

	**DM**				**NON-DM**			
	**IA**	**1 year**	**Diff %**	**P value**	**IA**	**1 year**	**Diff %**	**P value**
**Not smoking**	261/316 (82.6)	270/316 (85.4)	2.8%	0.035	455/703 (64.7)	479/703 (68.1)	3.4%	0.0003
**Oily fish target**	17/312 (5.4)	29/312 (9.3)	3.8%	0.008	34/700 (4.9)	84/700 (12.0)	7.1%	<0.001
**Fish target**	214/333 (62.3)	256/333 (76.9)	12.6%	<0.001	457/764 (59.8)	561/764 (73.4)	13.6%	<0.001
**F/V target**	190/316 (60.1)	262/316 (82.9)	22.7%	<0.001	329/703 (46.8)	532/703 (75.7)	28.9%	<0.001
**PA target**	95/312 (30.4)	206/312 (66.0)	35.6%	<0.001	205/698 (29.4)	536/698 (76.8)	47.4%	0.002
**Mean Steps/day**	6185	7294	1109	<0.001	6900	7984	1084	<0.001
**Ideal WC Females**	8/158 (5.1)	13/158 (8.2)	3.2%	0.025	83/338 (24.6)	97/338 (28.7)	4.1%	0.029
**Ideal WC Males**	30/146 (20.5)	39/146 (26.7)	6.2%	0.023	133/353 (37.7)	155/353 (43.9)	6.2%	0.005
**BMI target**	24/312 (7.7)	31/312 (9.9)	2.2%	0.06	169/697 (24.2)	196/697 (28.1)	3.9%	0.0001
**Mean weight**	83.6	82.5	−1.1	<0.001	78.7	77.6	−1.1	<0.001
**Mean TC**	5.48	5.11	−0.37	<0.001	6.14	5.63	−0.5	<0.001
**Mean LDL**	3.28	2.9	−0.38	<0.001	3.85	3.43	−0.42	<0.001
**Mean SBP**	140.6	133.9	−6.7	<0.001	141.4	133.6	−7.8	<0.001
**Mean DBP**	81.6	77.9	−3.7	<0.001	85.6	81.3	−4.2	<0.001
**TC target**	82/295 (27.8)	143/295 (48.5)	20.7%	<0.001	63/672 (9.4)	152/672 (22.6)	13.2%	<0.001
**LDL target**	93/274 (33.9)	157/271 (57.3)	23.4%	<0.001	96/652 (14.7)	196/652 (30.1)	15.3%	<0.001
**BP target (<130/85 DM; <140/90 Non-DM)**	76/311 (24.4)	120/311 (38.6)	14.1%	<0.001	286/698 (40.9)	467/698 (66.9)	25.9%	<0.001
**Mean HbA1c**	6.6	6.4	0.20	0.002	n/a	n/a	n/a	n/a
**BB**	62/129 (48.0)	62/129 (48.0)	0%	1.000	77/496 (15.5)	99/496 (19.9)	4.4%	0.0002
**ACEI/ARB**	128/184 (69.6)	131/184 (71.2)	1.6%	0.508	142/517 (27.5)	203/517 (39.3)	11.8%	<0.001
**Anti-platelet**	32/111 (28.8)	40/111 (36.0)	7.2%	0.008	50/483 (10.3)	71/483 (14.7)	4.3%	<0.001
**Statins**	73/164 (44.5)	102/164 (62.2)	17.7%	<0.001	77/542 (14.2)	200/542 (36.9)	22.7%	<0.001

**Figure 2:**
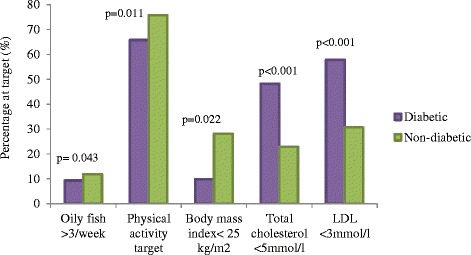
**Summary of significant differences among diabetic and non-diabetic high-risk individuals at one-year assessment.**

Data are proportion as a percentage % (of the numbers in each group who had both IA and one year data for the variables in question) and percent difference between IA and one year; ACEI/ARB- angiotensin converting enzyme inhibitor/angiotensin receptor blocker; BB- beta blocker; BMI- body mass index; CCB- calcium channel blocker; DBP- diastolic blood pressure; LDL- low density lipoprotein cholesterol; n/a- not applicable; SBP- systolic blood pressure; SF- saturated fat; TC- total cholesterol; TG- triglyceride.
- **Lipid and BP indices:** More patients with diabetes achieved the targets for TC (48.2% vs. 22.9%, p < 0.001), and LDL (57.9% vs. 30.7%, p < 0.001). Although there was a decrease in mean TC and LDL by one year in both groups of patients, the decrease was larger among the patients without diabetes (Table 5). Both groups had significant reductions in mean SBP and DBP and by one year, about two-thirds of patients in both groups achieved BP <140/90 mmHg (Table 5).
- **Drug therapy:** The proportion of patients with diabetes using all the classes of cardioprotective drugs was higher than the patients without diabetes but this was not significant after adjusting for the proportions achieving this target at baseline. Over two-thirds (70.3%) of the patients with diabetes used statins compared to only 40.3% of the patients without diabetes but this was not statistically significant (p = 0.088). While the use of all classes of cardioprotective medication increased significantly from baseline to one year among patients without diabetes, only antiplatelet and statin use increased significantly among patients with diabetes (Table 5). Figure 2 summarises the important differences at one-year.

### Glycaemic control among patients with diabetes

At baseline among coronary patients, the mean HbA1c was 6.84 ± 1.29% and this decreased slightly to 6.83 ± 1.30% (p = 0.845) by one year. The proportions achieving the glycaemic target (HbA1c < 7%) increased from baseline to one year but also not significantly. On the other hand, among HRIs, the mean HbA1c reduced significantly from a mean of 6.6 ± 1.3% at baseline to 6.4 ± 1.04% (p = 0.002) by one year and the proportions achieving the target HbA1c also improved significantly.

## Discussion

This study demonstrated that the proportion of diabetic and non-diabetic patients achieving the European lifestyle and risk factor targets for CVD prevention largely improved from baseline to one year (except for smoking among coronary patients). For dietary (except saturated fat among diabetics) and PA targets, there were significant improvements from baseline in both diabetic and non-diabetic coronary and HRIs. However, these improvements were less among the diabetics in the high-risk group. Non-smoking increased from baseline among HRIs (less among the diabetics) but reduced among coronary patients (more among diabetics). Murchie et al. showed that a nurse-led clinic for secondary prevention in coronary patients was effective in modifying several CVD risk factors but non-smoking remained unchanged at one year follow up [13]. The meta-analysis by Chow et al. illustrated that smoking cessation among CHD patients significantly reduces the risk of myocardial infarction by 43% [14]. However Janssen et al. found that the effect of lifestyle modification programmes on smoking cessation decreased with time [15]. One possible reason may be that having a cardiac event prompts quitting initially as a direct relation can be made to the smoking habit. However as time progresses some smokers will relapse, as smoking is a difficult addiction to break. This is worse among diabetics for whom the need to make multiple lifestyle changes may impact on their ability to make additional restrictions on their lifestyle [16]. This underlies the importance of assistance with pharmacotherapy in coronary patients trying to quit smoking as recommended [4].

Although a significant improvement from IA**,** the proportions of coronary diabetics and non-diabetics at oily fish target at one-year were low and not significantly different. Among HRIs however, significantly fewer diabetics achieved this target. A meta-analysis demonstrated that 1–2 servings of fish/week especially oily fish, is associated with a significant 36% and 17% reduction in coronary death and total mortality respectively, comparable to the protective effects of statins and is recommended by the European guidelines for primary and secondary CVD prevention [17,4]. However recent updated draft guidelines from the NICE suggests that this may only confer minimal additional benefits in preventing further events in CHD patients consequent to recent improvements in care and treatment [18].

Weight reduction contributes to reduction in BP, blood cholesterol and blood glucose [19]. Dietary modifications that contribute to weight loss include reduction of energy-dense saturated fat and increase in the intake of fruit and vegetables [20]. Although no significant differences were found in both groups of patients with regard to these factors, fewer coronary and HRIs with diabetes achieved BMI target and in fact, coronary diabetics gained weight. This is similar to the results obtained in a multicentre prospective study of multifactorial intervention in middle-aged T2DM patients where after one year of follow up, the intervention yielded significant improvements in several risk factors including BP and lipids but no effect on body weight [21].

Lower attainment of PA target among the diabetics (significantly among HRIs but not among coronary patients) may partly explain low achievement of BMI targets. Physical activity is an important non-pharmacologic tool for CVD prevention and optimum dose/effect benefit is obtained from PA levels with energy expenditure that corresponds to walking approximately 20–30 km/week at a speed of 4–5 km/h [22]. The guidelines recommend at least 150 min/week moderate aerobic physical activity that should be combined with three weekly sessions of resistance exercise to increase muscle strength [4]. A meta-analysis of 14 trials on the effect of PA on glycaemic control and BMI among diabetics found that it was beneficial in terms of HbA1c reduction but had no significant effect on body mass [23]. However, the Look AHEAD trial demonstrated that regular PA improved fitness and helped to sustain weight loss achieved by the study participants [24]. This may be due to the higher intensity of exercise prescribed in that trial compared with what was offered in EUROACTION, which was not equipment-based and was less intensive. Also many of the drugs used to control glycaemia lead to weight gain thus making weight loss among diabetics a difficult goal to attain [25].

Recent evidence has shown no additional mortality benefit from previous guideline recommendations of SBP below130 mmHg among diabetics, influencing the current conservative target below 140/85 mmHg [26-28]. Usually 2 or more drugs including any from the ACEI/ARB class are required to achieve BP control among diabetics [29]. Among both coronary and HRIs in this study, higher proportions of diabetics compared to the non-diabetics were on ACEI/ARBs (significantly among coronary patients). However among coronary patients, mean SBP increased and a lower proportion of the diabetics achieved BP <140/90 mmHg compared to non-diabetics (53.5% versus 74.0%, p < 0.001). Possible reasons could include among others, drug dosages and adherence, as it is likely that the diabetics may have been on many more medications. Many studies have shown that treatment compliance is usually poor among diabetics [30,31]. Data from EUROASPIRE III illustrated that obesity, DM and dyslipidaemia were predictors of poor BP control among CHD patients [32]. Poor attainments of BP goals among CHD patients have been reported in other studies [33,8]. Tranche et al. demonstrated that although only 24.5% of diabetics achieved BP <130/85 mmHg, improvement in BP control was the most significant contributor to reduced CVD risk [21].

At one year, over two-thirds of the coronary patients achieved the TC and LDL targets. The change in mean LDL and increase in proportion attaining LDL target was non-significant among diabetics but was significant among non-diabetics. This may be as a result of the significant increase in the use of statins among the non-diabetics, which was non-significant among the diabetics. The HRIs with DM were more likely to achieve both TC (48.2% vs 22.9%, p < 0.001) and LDL (57.9% vs 30.7%, p < 0.001) targets probably partly due to higher statin use. Still a reasonable proportion of these patients were above the targets and considering the benefits of lipid lowering on cardiovascular events, there is room for improvement [34]. Adherence to lifestyle modification including daily supplementation with functional foods like phytosterol-supplemented yogurt, was found in a multicenter cohort study to result in significant reductions in LDL (by 13.2%) in 1,048 HRIs, half of whom were already on statins. This is akin to what obtains with doubling the dose of statins without the problem of additional side effects although as no clinical trials on cardiovascular endpoints have been done, it is not a firm recommendation in the guidelines [35,4].

The efficacy of cardioprotective medications used in this study for secondary prevention is well established and recommended [4]. As was evident from the three EUROASPIRE surveys their use has increased with time [8]. This present study found that majority of the coronary patients used all classes of cardioprotective medication (significant increase from baseline for ACEI/ARB in both diabetics and non-diabetics and statins among non-diabetics only). Medication use was lower among the HRIs where a non-significantly higher proportion of diabetics used them compared to non-diabetics. Current evidence is not in favour of the use of antiplatelet treatment for primary prevention in DM. De Berardis et al., in their meta-analysis of six RCTs found no statistically significant reduction in the risk of major cardiovascular events or all-cause mortality obtained from low-dose aspirin use compared to placebo in diabetics without established CVD [36]. Among HRIs, use of anti-platelets was relatively low but increased significantly in both groups from baseline. Possible reasons may include other indications for its use such as in hypertensive individuals with renal impairment or at high-CVD risk, but the retrospective nature of this study precludes these assumptions.

### Glycaemic control

The evidence from STENO-2 and the relatively low event rates in ACCORD, ADVANCE, and VADT indicates that in T2DM, control of other non-glycaemic risk factors with lifestyle modification and cardioprotective medication may be more beneficial in terms of CVD outcomes than glycaemic control alone [37]. In the present study, diabetics received such a multifactorial intervention and the proportions achieving glycaemic targets improved from baseline. An interesting finding that was not a pre-specified outcome, is the higher proportion of diabetics in the high-risk group (79.1%) meeting HbA1c targets compared to coronary diabetics (59.4%). Possible explanations include that coronary patients may have a longer duration of diabetes, be on other interacting medications or have contraindications to particular hypoglycaemic drugs. This requires further research although similar results were demonstrated in a European study where more diabetics without CVD achieved better glycaemic control over a 4-year period compared to diabetics with CVD [38].

### Study strengths and limitations

The strength of this study include that the participants were managed in busy general hospitals and general practices, demonstrating the feasibility of the application of this intervention in everyday clinical practice as evidence shows that when risk is modified in diabetics, they achieve greater CVD benefits than their non-diabetic counterparts [39].

This is a sub-group analysis of a RCT that was not pre-specified. Therefore it is underpowered and the results demonstrating differences between the diabetics and non-diabetics cannot be over-interpreted and at best should be viewed as hypothesis generating [40]. Furthermore, the findings from this study are only informative and no definite conclusion can be drawn about whether these differences were the effect of the EUROACTION intervention, as the usual care arm of the trial was not analysed.

## Conclusion

This study showed that diabetics improved their risk factors similar to non-diabetics but there were significant differences especially with regards to lifestyle targets. With regards to BP control, fewer patients with diabetes who had suffered a coronary event achieved this target. All confounding factors could not be adjusted for in this retrospective study therefore, based on these results, a prospective randomised study will be better to determine the factors influencing these differences among diabetics and non-diabetics. Further research is needed to determine effective means of translating evidence to clinical practice to assist diabetic individuals modify their risk.

## Additional files

Additional file 1: Table S1. European lifestyle, risk factor and therapeutic targets (From Wood et al., 2004 with permission). Additional file 2: Table S2. Primary outcomes.
