# Supplementary material for: Comparison of treatment outcomes in patients with and without diabetes mellitus attending a multidisciplinary cardiovascular prevention programme (a retrospective analysis of the EUROACTION trial)
Source: BMC Cardiovasc Disord. 2015 Feb 24;15:11. doi: 10.1186/s12872-015-0006-4 (PMC4356146; doi:10.1186/s12872-015-0006-4)
Supplement: Additional file 1: Table S1. — European lifestyle, risk factor and therapeutic targets (From Wood et al., 2004 with permission). [file 12872_2015_6_MOESM1_ESM.doc]

**Additional file 1**

**Table S1.** European lifestyle, risk factor and therapeutic targets (From Wood et al., 2004 with permission)

| - Giving up smoking |
| --- |
| - Eating a healthy diet |
| - Becoming physically active |
| - Achieving and maintaining a healthy shape (waist circumference below 94 cm {below 37 inches} for men and below 80 cm {below 31.5 inches} for women) and weight (Body Mass Index below 25kg/m2) |
| - Blood pressure below 140/90 mmHg (for those with diabetes below 130/80 mmHg) |
| - Total cholesterol below 5.0 mmol/l; LDL cholesterol below 3.0 mmol/l |
| - Blood glucose below 6.1 mmol/l (below 110 mg/dl) and good glycaemic control in all persons with diabetes |
| - To ensure that each of the following classes of cardio-protective medications are prescribed as clinically indicated, at the doses used in the clinical trials, for all coronary patients and to ensure long-term compliance with these therapies:   -Antiplatelet therapies  -Beta blockers  -Angiotensin converting enzyme inhibitors/angiotensin II receptor blockers (ACEI/ARB) and  -Lipid lowering therapy (statins) |
