# Supplementary material for: Comparison of treatment outcomes in patients with and without diabetes mellitus attending a multidisciplinary cardiovascular prevention programme (a retrospective analysis of the EUROACTION trial)
Source: BMC Cardiovasc Disord. 2015 Feb 24;15:11. doi: 10.1186/s12872-015-0006-4 (PMC4356146; doi:10.1186/s12872-015-0006-4)
Supplement: Additional file 2: Table S2. — Primary outcomes. [file 12872_2015_6_MOESM2_ESM.doc]

Additional file 2

**Table S2. Primary outcomes**

| **RISK FACTOR** | **TARGET^$^** |
| --- | --- |
| Smoking | Quit smoking (breath carbon monoxide <6 parts per million) |
| Diet   - Saturated fat* - Fruit and vegetables - Oily fish - Fish | <10% total daily energy intake  >400g daily  >3 times a week  >20 g/day |
| Anthropometry   - Body mass index - Waist circumference | <25kg/m^2^  ≤80cm (females), ≤94cm (males) |
| Physical activity | 30-45 minutes moderate intensity 4-5 times a week |
| Blood pressure | <140/90 mmHg (non-diabetics), <130/85 mmHg (diabetics and coronary patients) |
| Blood cholesterol | Total cholesterol <5.0 mmol/l  LDL cholesterol <3.0 mmol/l |
| Blood glucose and diabetes | Glycated haemoglobin (HbA1c) <7% |
| Cardioprotective drugs (antiplatelet, B blockers, angiotensin-converting enzyme inhibitors or angiotensin II receptor blockers and statins) | As clinically indicated, at evidence-based doses. |

*Only in the hospital patients; **^$^**these targets are same as those used in Wood et al., 2008.
